# Establishment of a Cell Model for Dynamic Monitoring of Intracellular Calcium Concentration and High-Throughput Screening of P2Y2 Regulators

**DOI:** 10.3390/molecules27093003

**Published:** 2022-05-07

**Authors:** Mingda Wu, Cheng Hu, Jingsong Liu, Chuannan Wu, Xueying Liu, Feng Hao, Wenliang Li

**Affiliations:** 1 College of Laboratory Medicine, Jilin Medical University, Jilin 132000, China; 2019010438@ybu.edu.cn (M.W.); chenghu122@sina.com (C.H.); 2 Medical College, Yanbian University, Yanji 136200, China; 3 Department of Thoracic Surgery, The Affiliated Hospital of Xuzhou Medical University, Xuzhou 221000, China; jingsong678@sina.com; 4 The Municipal Hospital Affiliated to Xuzhou Medical University, Xuzhou 221000, China; chuannan4026@sina.com; 5 School of Medical Technology, Beihua University, Jilin 132000, China; liuxueying0220@sina.com; 6 Jilin Collaborative Innovation Center for Antibody Engineering, Jilin Medical University, Jilin 132000, China

**Keywords:** P2Y2, GPCRs, regulators, high-throughput screening, calcium concentration

## Abstract

P2Y receptors are G-protein-coupled receptors (GPCRs) for extracellular nucleotides. The P2Y2 receptor subtype is expressed in a variety of cell types and plays an important role in physiological and pathophysiological processes such as inflammatory responses and neuropathic pain. Based on this, the P2Y2 has been identified as an important drug target. The specificity of current P2Y2 receptor modulators is relatively poor, and currently, specific and efficient P2Y2 receptor modulators and efficient screening strategies are lacking. In this study, a cell model based on calcium-activated chloride channels (CaCCs) was established that can detect changes in intracellular calcium concentrations and can be used to high-throughput screen for P2Y2 receptor-specific regulators. This screening strategy is suitable for screening of most G-protein-coupled receptor regulators that mediate increases in intracellular calcium signals. The cell model consists of three components that include the endogenously expressed P2Y2 receptor protein, the exogenously expressed calcium-activated chloride channel Anoctamin-1 (Ano1), and a yellow fluorescent protein mutant expressed within the cell that is highly sensitive to iodine ions. This model will allow for high-throughput screening of GPCR regulators that mediate increased intracellular calcium signaling using the calcium-activated transport of iodide ions by Ano1. We verified the ability of the model to detect intracellular calcium ion concentration using fluorescence quenching kinetic experiments by applying existing P2Y2 agonists and inhibitors to validate the screening function of the model, and we also evaluated the performance of the model in the context of high-throughput screening studies. The experimental results revealed that the model could sensitively detect intracellular calcium ion concentration changes and that the model was accurate in regard to detecting P2Y2 modulators. The resultant value of the Z-factor was 0.69, thus indicating that the model possesses good sensitivity and specificity.

## 1. Introduction

P2Y receptors are extracellular nucleotide-activated G-protein-coupled receptors (GPCRs). There are eight isoforms of mammalian P2Y receptors that are divided into two subtypes (P2Y1, P2Y2, P2Y4, P2Y6, P2Y11, P2Y12, P2Y13, and P2Y14). P2Y2 receptors, a subtype of these receptors, are widely distributed in various human cells, particularly in immune cells, epithelial and endothelial cells, renal tubules, and osteoblasts, and they play important roles in physiological responses such as ion channel regulation, cell proliferation regulation, and cell cycle regulation [1,2]. Notably, P2Y2 is closely associated with epithelial chloride secretion, which is an important target for the treatment of cystic fibrosis, and activation of the P2Y2 receptor increases epithelial chloride secretion and inhibits sodium uptake by epithelial cells, thus promoting water secretion and mucin release in the airway epithelium and ocular epithelium in individuals with strong associations with lung and eye disease release [3,4,5]. Additionally, the P2Y2 receptor, when coupled to the G-proteins Gq and G11, plays a key role in fluid shear stress-induced activation of endothelial nitric oxide synthase (eNOS) that controls the vascular tone and blood pressure [6]. The ATP-activated endothelial P2Y2 receptor is also a major mediator of platelet activation by tumor cells, and this in turn promotes the survival and spread of tumor cells. Specific inhibitors of P2Y2 are expected to be useful as new drugs for antimetastatic therapy in the context of various tumors [7]. The lack of selectivity of most currently developed P2Y2 receptor agonists indicates that they interact with P2Y2 receptors while activating other P2Y receptors, such as P2Y4 receptors and P2Y6 receptors [8,9]. Therefore, screening for P2Y2 receptor-specific modulators is particularly important.

GPCRs are the largest and most versatile class of cell surface receptors and are an important target for the identification of clinically useful agonists and antagonists, where they account for 15% of the total “available” genome and 25% of marketed drugs [10]. The primary approach involves detecting changes in the concentrations of intracellular second messengers such as small molecules that bind to G-protein-coupled receptors and trigger an increase in intracellular calcium or cAMP concentrations due to intracellular signaling to ultimately verify those small molecules are agonists of G-protein-coupled receptors. The primary method that is currently used for intracellular calcium detection involves the use of a calcium fluorescent probe such as the commonly used Fluo-2 AM and the modified upgraded Fluo-4 AM [11,12]. The limitations of such probes in regard to handling and cell loading prevent their application in high-throughput drug screening.

**Figure 1:**
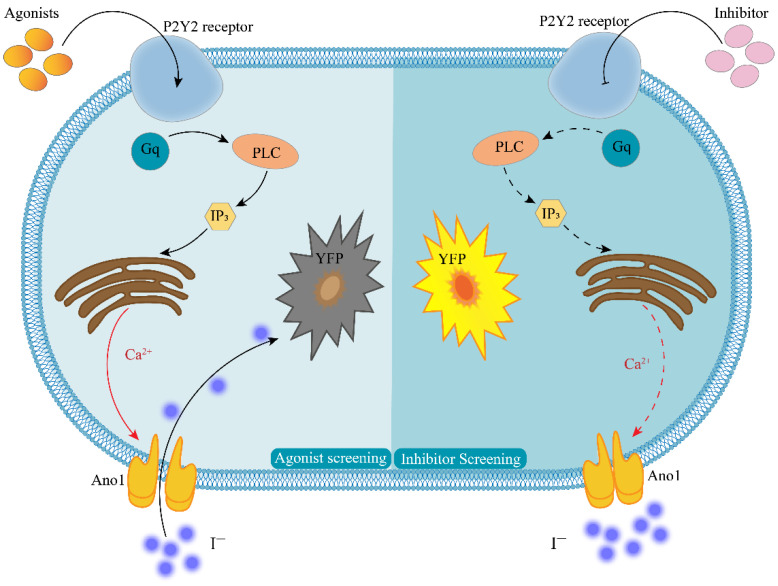
Principle of cell model screening for P2Y2 modulators. The left half of the figure shows the agonist screening strategy, and the right half shows the inhibitor screening strategy; Agonists—P2Y2-specific agonists; Inhibitor—P2Y2-specific inhibitors; P2Y2—purinergic receptors P2Y2; Gq—G-protein subunit; PLC—phospholipase C; IP_3_—inositol trisphosphate; YFP—yellow fluorescent protein mutant H148Q/I152L; Ca^2+^—calcium ion; I^−^—iodine ion; Ano1—calcium-activated chloride channel, Anoctamin-1.

In this study, we report an efficient assay for detecting GPCR activity for Gq-coupled GPCRs in which Ca^2+^ is assayed from the halide conductance of the Anoctamin-1 (Ano1) protein. The membrane protein Ano1 and the intracytoplasmic yellow fluorescent protein mutant (YFP-H148Q/I152L) were overexpressed in Fischer rat thyroid (FRT) cells that endogenously express P2Y2 receptors. Ano1, a calcium-activated anion channel, is a powerful transporter of iodide ions, where a single channel is capable of reaching 10^6^ orders of magnitude per second. Additionally, Ano1 is sensitive to intracellular calcium ion concentrations in response to intracellular calcium ion concentrations at the nanomolar level [13]. The intracellularly expressed YFP-H148Q/I152L was highly sensitive to iodine ions, and 1.9 mM of iodine ions quenched 50% of the fluorescence intensity of YFP-H148Q/I152L [14]. The specific working principle is presented in Figure 1. When a P2Y2-specific agonist binds to the P2Y2 receptor, intracellular signal transduction occurs. This results in the release of calcium from the calcium pool of the endoplasmic reticulum, and this in turn agonizes the Ano1 calcium-activated anion channel. At this time, PBS-containing sodium iodide is added, and the rapid inward flow of iodine ions induces the quenching of YFP-H148Q/I152L. The degree of quenching is positively correlated with the degree of opening of Ano1. Thus, the model could also respond to changes in intracellular calcium ion concentration. When screening for P2Y2-specific inhibitors, the small molecule to be tested is first incubated with the cell model and then sodium iodide PBS-containing ATP is added; if the fluorescence is not quenched, the small molecule is likely to be an inhibitor of the P2Y2 receptor.

## 2. Results

### 2.1. Establishment of FRT Cell Model Stably Expressing Ano1, YFP-H148Q/I152L, and Endogenous P2Y2

**Figure 2:**
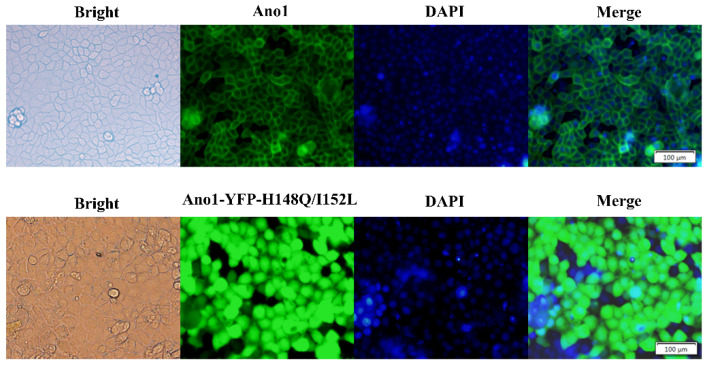
Fluorescence images of FRT cells transfected with Ano1 and Ano1-YFP. Fluorescence images revealed that Ano1 was distributed in the plasma membrane and that YFP-H148Q/I152L was expressed in the cytoplasm.

Stably transfected FRT cell lines were generated that co-expressed Ano1 and YFP-H148Q/I152L and endogenously expressed wild-type P2Y2. Ano1 fusionally expressed GFP and exhibited a plasma membrane distribution according to fluorescence microscopy images. FRT cells expressing Ano1 were transfected with YFP-H148Q/I152L, and fluorescence microscopy images revealed that YFP-H148Q/I152L was stably expressed inside the cells (Figure 2).

**Figure 3:**
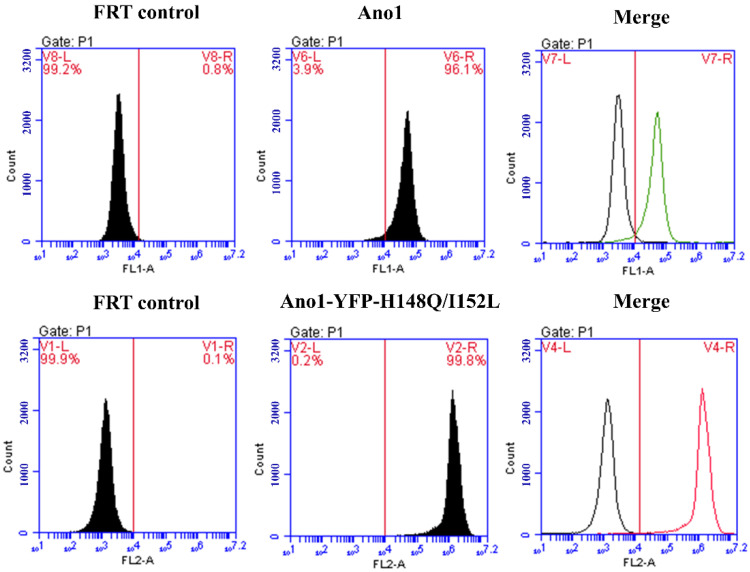
Flow cytometry detection of Ano1 and Ano1-YFP-H148Q/I152L stably transfected cells purity. FRT control group is untransfected FRT cells.

The results of flow cytometry analysis demonstrated that the FRT cell lines expressing Ano1 and co-expressing Ano1 and YFP-H148Q/I152L both possessed a stable transformation efficiency of more than 95% (Figure 3). Additionally, the fluorescence signal intensity was high and could be used for subsequent detection.

### 2.2. Endogenous Expression of P2Y2 in FRT Cells

**Figure 4:**
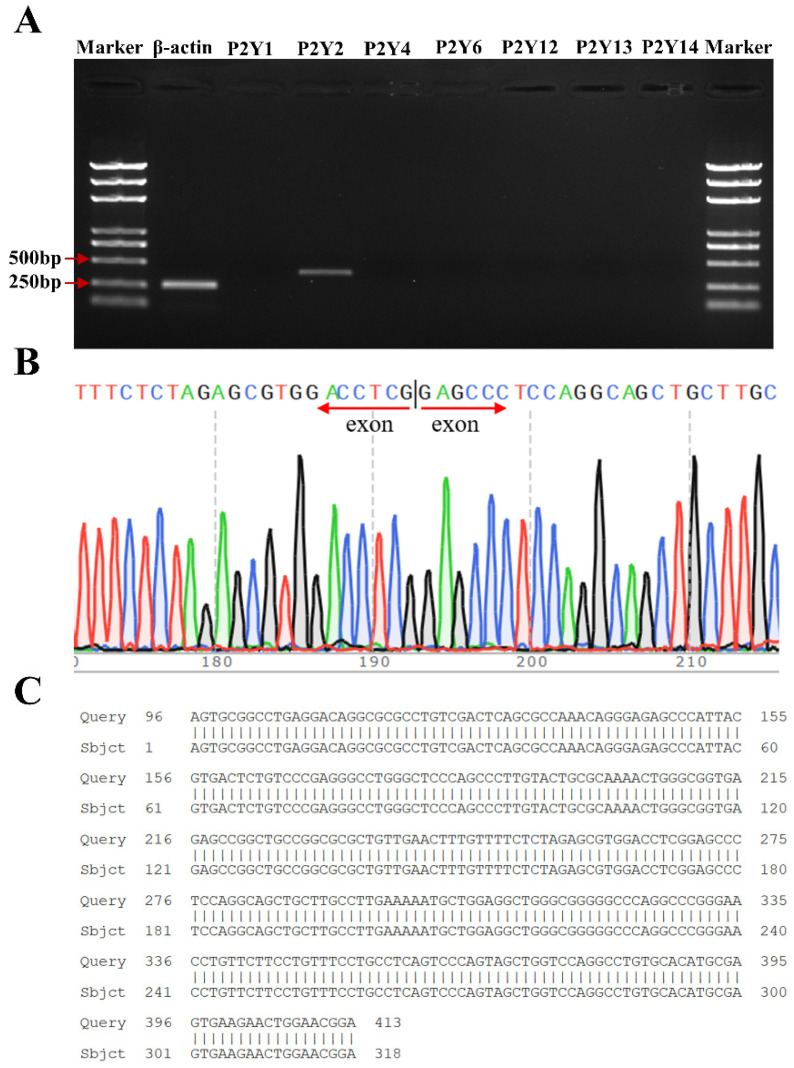
Confirmation of the endogenous expression of P2Y2 in FRT cells. (**A**) RT-PCR verification of P2Y2 mRNA levels. (**B**) Sequencing results for the PCR products of P2Y2. (**C**) Comparison of P2Y2 sequencing results to the P2Y2 gene sequence.

RT-PCR analysis revealed specific bands at 365 bp and 260 bp specific for P2Y2 and β-actin, respectively, and these bands were consistent with the size of the previously designed P2Y2-specific primer amplification products. P2Y1, P2Y4, P2Y6, and P2Y12–14 did not amplify any specific bands (Figure 4A), thus demonstrating that FRT cells express P2Y2 at the mRNA level. The sequencing results were analyzed using Chromas software, the nucleotide sequences were compared using NCBI-BLAST, and the results were completely consistent with the gene sequence of P2Y2 (NM_017255.2) in the GenBank database, thus indicating that the replicated DNA fragment was the target gene (Figure 4B,C).

**Figure 5:**
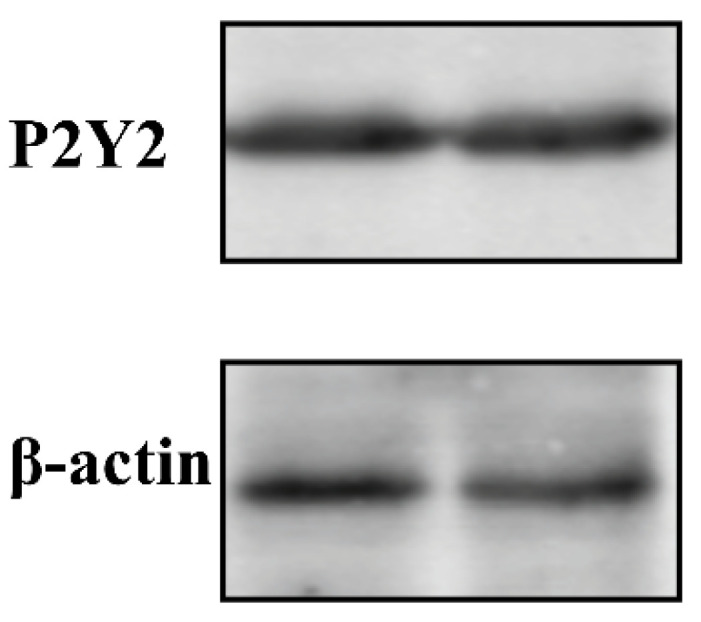
Western blot analysis of P2Y2 in FRT cell lines. Whole-cell lysates that equal amounts of total protein (30 µg) were separated on 10% sodium dodecyl sulfate-polyacrylamide gels, subjected to western blotting, and probed with the P2Y2-rabbit or β-actin mouse primary antibodies. Then, PVDF membranes were followed by exposure to different fluorescently labeled secondary antibodies. Immunoreactive proteins were visualized using an Odyssey infrared imaging system (Li-Cor). Western blotting was repeated at least three times, and one or two representative blots are presented.

To further confirm the endogenous expression of P2Y2 in FRT cells, we measured the protein levels. Western blot results revealed that P2Y2 and β-actin exhibited a specific band at 42 kDa. These results indicate that FRT cells express the P2Y2 protein (Figure 5).

### 2.3. Functional Validation of the “Detection Element” in the Cell Model

In this cell model, Ano1 acts as a detection element in response to intracellular Ca^2+^ signals and must exhibit calcium concentration-dependent opening properties, and YFP-H148Q/I152L should exhibit sensitivity to iodine ions that are transported by the activated opening of Ano1 channels.

**Figure 6:**
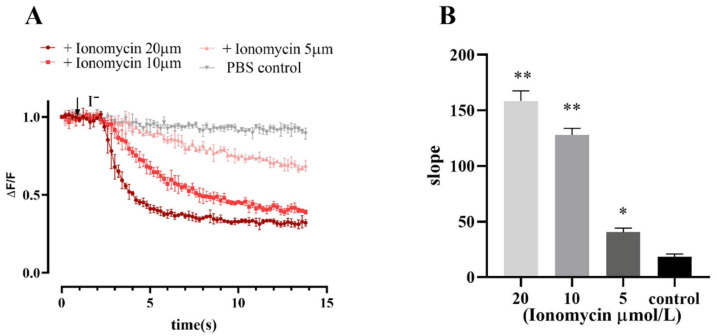
Fluorescence quenching kinetic experiment results verified the functionality of Ano1 and YFP-H148Q/I152L in FRT cells. (**A**) Ionomycin dose-response study; values were obtained as difference of each individual point with first recorded value of the experiment (ΔF) divided per first recorded value (F). (**B**) The slope of the fluorescence quenching curve presented in A. Data represent the mean ± SD (*n* = 3 independent experiments). ** *p* < 0.01, * *p* < 0.05 versus control group.

Ionomycin is often used as a calcium carrier in experiments to increase intracellular Ca^2+^ levels across various biological membranes. The results of fluorescence quenching kinetic experiments revealed that the cell model responded to different concentrations of ionomycin, and the slope value of the induced fluorescence quenching curve increased with increasing ionomycin concentration (Figure 6).

**Figure 7:**
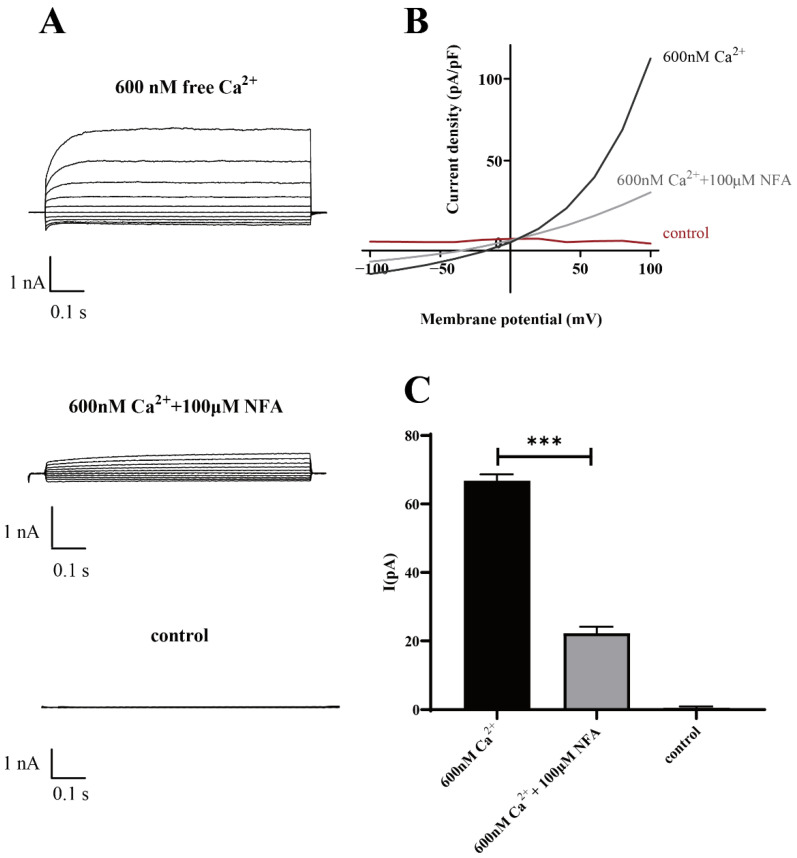
Patch-clamp analysis of Ano1 inhibition by NFA in FRT-Ano1 cells. (**A**) Whole-cell Ano1 current was recorded at a holding potential of 0 mV and was pulsed to voltages between ± 100 mV (in steps of 20 mV) in the absence and presence of 100 µM NFA. Ano1 was activated by 600 nM Ca^2+^. (**B**) Steady-state current-voltage relationships of currents at 600 nM Ca^2+^, 100 µM NFA, and control. (**C**) The bar graphs summarize the current density data measured at +80 mV (mean ± SD., *n* = 3). *** *p* < 0.001.

The results of the patch-clamp data demonstrated that Ano1 could be directly activated by calcium ions, and at the same calcium activation concentration, the Ano1 inhibitor NFA can effectively inhibit the open current of Ano1 (Figure 7).

The above results confirm that Ano1 can be activated by intracellular calcium ions and that YFP-H148Q/I152L can directly respond to the channel opening degree by quenching the degree of sensitivity to transport iodide ions and laterally respond to changes in intracellular calcium ion concentration.

### 2.4. Verification That the Cell Model Can Be Used to Detect Intracellular Calcium Ion Concentration

**Figure 8:**
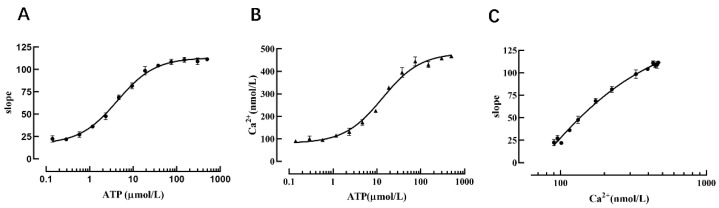
Verification that the cell model can reflect intracellular calcium concentration. (**A**) Dose-dependent curve of ATP and the slope value. (**B**) Dose-dependent curve of ATP and the intracellular calcium ion concentration. (**C**) Dose-dependent curve of the intracellular calcium ion concentration and the slope value.

Changes in calcium ion concentration are important modes of information transmission for cascade responses in living cells after receiving external stimuli; therefore, intracellular Ca^2+^ detection is a very important research tool in the context of signal transduction in G-protein-coupled receptor-mediated screening of relevant drugs and other related experiments. To confirm that the cell model possesses the function of detecting intracellular calcium ion concentration, we used different concentrations of ATP (an agonist of the P2Y2 receptor). After the P2Y2 receptor was activated, calcium ions in the endoplasmic reticulum were released through intracellular signal transduction, and the Ano1 channel was activated to cause iodine ions to flow inward, thus resulting in YFP-H148Q/I152L fluorescence quenching. The results revealed that the slope value of the fluorescence quenching curve was increased with increasing concentrations of ATP (Figure 8A).

Concurrently, the calcium-sensitive fluorescent indicator Fura-2 was used to detect changes in cellular calcium concentration triggered by different concentrations of ATP-activated P2Y2 receptors, the results showed that the intracellular calcium ion concentration was increased with an increase in ATP concentration (Figure 8B), and the data analysis indicated that the slope value of the fluorescence quenching curve exhibited a dose-dependent relationship with calcium ions (Figure 8C). Therefore, one slope value corresponds to one intracellular calcium ion concentration. When measuring intracellular calcium ion concentration using this method, it is only necessary to perform a fluorescence quenching kinetic experiment to calculate the slope value of the fluorescence quenching curve, and then the intracellular calcium ion concentration can be deduced from the slope value of the fluorescence quenching curve and the standard curve of calcium ion concentration.

### 2.5. Functional Validation of High-Throughput Screening of P2Y2 Modulators

Currently established P2Y2 receptor activators and inhibitors were selected to validate that the model could be applied to the screening of P2Y2 receptor modulators. Three P2Y2 receptor activators (ATP, ADP and UTP) were selected to validate the model for the screening of P2Y2 receptor modulators, and the slope values of the fluorescence quenching curves were assessed by applying the same concentration gradient.

**Figure 9:**
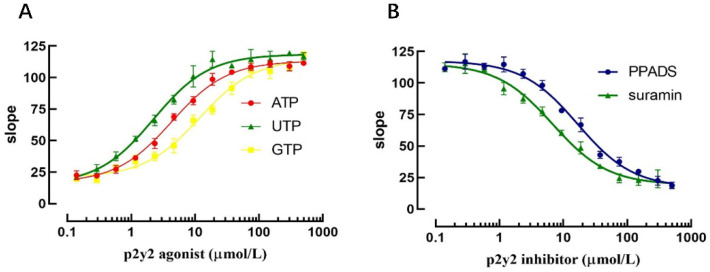
Validation of the screening assay for identification of P2Y2 regulators. (**A**) Validation of P2Y2 agonist screening. (**B**) Validation of P2Y2 inhibitor screening.

The results revealed that at higher agonist concentrations, the corresponding slope value increased accordingly and exhibited a good dose-dependent relationship. The EC_50_ values for ATP, ADP, and UTP were 4.32 μmol, 2.23 μmol, and 11.82 μmol, respectively, according to the dose-dependent curve. Similarly, after applying the P2Y2 receptor inhibitor for 10 min, sodium iodide PBS-containing agonist was added, and the results revealed that the slope value of the fluorescence quenching curve was decreased with increasing inhibitor concentration. The IC_50_ values of the P2Y2 receptor inhibitors PPADS and Suramin were 16.81 μmol and 6.80 μmol, respectively, according to the dose-dependent curve (Figure 9).

The above results indicate that the model can not only screen P2Y2-specific regulators but can also evaluate the efficiency of different regulators.

**Figure 10:**
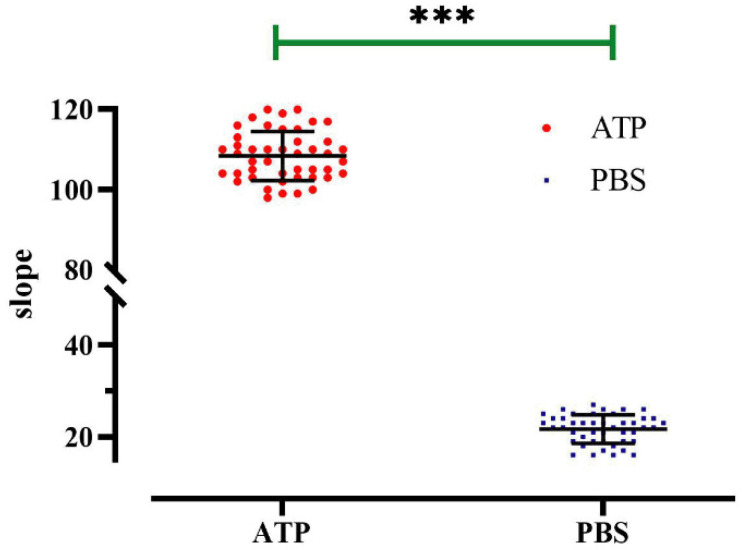
Z-factor evaluation of a high-throughput screening model for P2Y2 modulators. Slope value detected in each well of a 96-well plate; data represent the mean ± SD (*n* = 45 independent experiments). *** *p* < 0.001 versus PBS group.

The applicability of the method in the context of high-throughput screening was evaluated by experimentally measuring the Z-factor, a quantitative indicator of “goodness” that depends upon the difference between positive and negative control signals and their standard deviation. ATP was used as the positive control, and PBS was used as the negative control. A 96-well cell culture plate was used as a high-throughput screening unit, and the fluorescence quenching curve of each well was detected after 20 min of incubation. The results revealed well-separated positive and negative controls (Figure 10), and analyzing and calculating of the 96-well data revealed that Z-factor = 0.69. A Z-factor greater than 0.5 is typically considered to be excellent, and based on this, we concluded that this cell model possessed high sensitivity in regard to screening for regulators.

## 3. Discussion

P2Y2, an important isoform of P2Y, is coupled to the G-protein Gq/11 and its signal acts on PIP2 in the cell membrane via PLC to hydrolyze it to IP_3_ and DG. IP_3_ causes Ca^2+^ release from the endoplasmic reticulum calcium pool and activates PKC, and this in turn phosphorylates various intracellular cytokines to produce a variety of physiological effects [15]. Several studies have identified P2Y2 receptors and their signaling pathways as potential pharmacological targets for inflammation, atherosclerosis, cystic fibrosis, eye disease, and neurodegenerative diseases [3,4,5,16]. In addition, it has been reported that ATP and UTP at low concentrations strongly inhibit bone formation by osteoblasts in vitro through activation of P2Y2 receptors; thus, which role P2Y2 receptors play in the bone healing process deserves further investigation [17,18]. The commonly used P2Y2 agonists include UTP, ATP, ADP, and 2-MeSATP, and the antagonists include suramin and PPADS [15]. Among them, UTP cannot be used as a therapeutic agent for P2Y2-related diseases, as it is rapidly metabolized within human tissues. Suramin and PPADS are both antagonists of P2Y receptors, and PPADS exerts a relatively weak antagonistic effect on P2Y2. The P2Y2 modulators discovered in recent years almost all act on other subtypes of the P2Y receptor, and there is a lack of highly specific and high-affinity receptor agonists and antagonists for P2Y2. Based on this, effective screening techniques for P2Y2 receptor-specific modulators are urgently required.

Here, we report a high-throughput screening model for P2Y2 modulators based on Ano1 calcium-activated chloride channels that use FRT cells as a carrier and Ano1 as a detection element for calcium ions, and this model can respond to different concentrations of intracellular calcium ions. The fluorescent signal of YFP-H148Q/I152L, used as the detection signal, can respond to the degree of activation of Ano1 by different concentrations of intracellular calcium ions. The selected FRT cell line is equally important as a carrier of the assayed protein and the above two reaction elements. The use of this cell line for high-throughput drug screening has been well validated. In general, the selected FRT cell line possesses several advantages that include rapid growth on relatively inexpensive media, good adhesion to plastic multi-well plates during the solution wash exchange, stable expression of transfected proteins, and formation of tight junctions.

Functional assays for cell responses are widely used in high-throughput screening, in part because they are technically easier than fluorescent probe binding assays. The binding test cannot distinguish agonists and antagonists, and the detection sensitivity of allosteric regulators is low. For example, the Fluo-4 AM fluorescent probes currently used in most laboratories to detect calcium ions must be incubated for at least 30 min prior to detection, and there are some differences in the capacity of different cells to load them. Additionally, the price can reach $600 (U.S.). We propose a high-throughput screening model of Gq-protein-coupled GPCR regulators based on Ano1 that possesses several advantages, including (1) a strong and easily captured optical signal of the YFP-H148Q/I152L fluorescent protein, (2) the requirement of only a trace amount of intracellular calcium signal to activate Ano1 that allows for the detection of linear changes in fluorescence signal strength, enabling quantitative visualization, (3) the ability to provide high extracellular iodine concentrations artificially with a controlled fluorescence quenching response to easily provide a good negative control (an activator in the absence of extracellular iodine environment and channel activation where the fluorescence is not quenched), (4) a relatively less time-consuming and highly reproducible process during high-throughput screening with a measured Z-factor ≥0.6 that indicates a very good sensitivity and specificity in a single preliminary screening, and (5) good cell retention relative to traditional fluorescent probe methods, where cell lines stably expressing fluorescent proteins can be propagated and amplified without additional probe costs, thus making the process more economical and convenient.

Our assay requires a specialized cell line expressing Ano1, YFP, and the GPCR of interest, and theoretically, any GPCR that mediates elevated intracellular calcium signaling can be stably expressed in FRT cells by exogenous gene transfer, thus allowing this model to perform a high-throughput screening for regulators of the target GPCR. Our experiments were designed as a “pathway screen” to allow compounds that inhibit Ano1 iodine influx to be predicted to function as P2Y2 antagonists, Gq inhibitors, IP_3_ receptor inhibitors, or Ano1 inhibitors. This pathway screen possesses the advantage of identifying multiple targets of small molecule modulators on a single screen, whereas target identification is performed in a small secondary assay.

In summary, we have successfully constructed a new high-throughput screening model for Gq-protein-coupled GPCR regulators that allows for the dynamic detection of intracellular calcium ion concentration changes. The method is highly sensitive, technically simple, and inexpensive, and it can be used for high-throughput drug screening experiments, thus laying the foundation for the discovery of novel modulators of GPCR.

## 4. Materials and Methods

### 4.1. Materials

The RT-PCR kit (Takara Bio, Dalian, China), PCR kit, DNA Marker (Transgen, Beijing, China), agarose (OXOID, Thermo Fisher, Shanghai, China)), RIPA, DAB color development solution (Beyotime, Shanghai, China), rabbit anti-rat P2Y2 polyclonal antibody, rabbit anti-rat β-actin monoclonal antibody, and goat anti-rabbit IgG (Abcam, Shanghai, China) were all acquired from commercial sources. The Lipofectamine 3000 transfection reagent, puromycin antibiotic, bacteriostat antibiotic (Invitrogen, Carlsbad, CA, USA), Fura-2/AM, ATP, UTP, ADP, suramin, PPADS, ionomycin, and NFA (Sigma-Aldrich, Shanghai, China) were purchased from various vendors.

### 4.2. Cell Culture and Plasmid Construction

Fisher rat thyroid (FRT) and human embryonic kidney (HEK293T) cell lines were donated by professor Tonghui Ma of Northeast Normal University. FRT cells were cultured in modified F12 medium that was supplemented with 10% fetal bovine serum (FBS) (Gibco, Thermo Fisher, Shanghai, China), 2 mM L-glutamine, 100 U/mL penicillin, and 100 µg/mL streptomycin. HEK293T cell lines were cultured in RPMI 1640 medium containing 10% fetal bovine serum, 2 mmol/L L-glutamine, 100 U/mL penicillin, and 100 µg/mL streptomycin.

For expression in mammalian cells, a DNA fragment encoding a mouse Ano1-green fluorescent protein (GFP) fusion protein and the halide sensor YFP-H148Q/I152L were subcloned into plvx/blasticidin and plvx/puromycin lentiviral overexpression vectors.

### 4.3. Production of Lentivirus Particles and Transduction of FRT Cells

Lentiviral production and transduction were performed as described previously [19]. Briefly, HEK293T cells were co-transfected with pMD-VSVG, pCMV-delta R8.2 (Addgene, Beijing, China), and a lentiviral vector loaded with the target gene using polybrene [20]. Virus-containing medium was harvested at 48 h after transfection. FRT cells were infected with 1 mL of virus supernatant and 4 μg/mL of polybrene (Sigma-Aldrich, Shanghai, China) and then further cultured for 48 h before screening for overexpression monoclonal clones expressing resistance through the use of 0.5 μg/mL of puromycin (YFP-H148Q/I152L high-expression lentiviral vector) or 50 μg/mL of blasticidin (Ano1 high-expression lentiviral vector). In this experiment, the monoclonal overexpression vector of Ano1 was preferentially constructed, and the Ano1 monoclonal cells were expanded and cultured. They were then screened with YFP-H148Q/I152L high-expression lentivirus after infection to obtain the final co-transformed Ano1 and YFP-H148Q/I152L FRT cell line.

### 4.4. Detection of Endogenous Expression of P2Y2 in FRT Cells by RT-PCR

The total RNA was first extracted from FRT cells using the TRIzol method on ice as follows. FRT cells (1–5) × 10^6^·mL^−1^ were harvested, 1 mL of TRIzol was added to lyse the cells, and 0.2 mL of chloroform was added and then shaken and mixed. The mixtures were then centrifuged at 10,000× *g* for 15 min at 4 °C. The RNA was washed with 75% ethanol in DEPC water (7500 g) and centrifuged at 4 °C for 5 min. After drying at room temperature, 20 μL of DEPC water was added to obtain the RNA solution. The RNA concentration and purity were measured using a Nanodrop 2000 (Thermo Scientific, Shanghai, China), and the RNA solution was subjected to agarose gel electrophoresis to assess the RNA integrity. The cDNA solution was then reverse transcribed using a reverse transcription kit. Five pairs of specific primers were designed and synthesized (Table 1). The cDNA was used as the template for the PCR, and the products were subjected to agarose gel electrophoresis. The target bands were recovered by gel cutting using the SanPrep Column DNA Gel Extraction Kit (Sangon Biotech, Shanghai, China). The recovered DNA solution was subjected to nucleic acid sequencing (Sangon Biotech, Shanghai, China).

### 4.5. Western Blot Analysis

Whole-cell lysates were prepared by sonication in 10 mmol/L Tris-Cl (pH 7.0) containing 1% SDS, protease inhibitors, and phosphatase inhibitors (Roche Diagnostics, Shanghai, China). Protein concentration was determined using a bicinchoninic acid protein assay kit(Thermo Fisher, Shanghai, China). Samples were separated by SDS-polyacrylamide gel electrophoresis and transferred to polyvinylidene difluoride membranes (Thermo Fisher, Shanghai, China). After blocking for 1 h at room temperature in TBS buffer (150 mmol/L NaCl, 10 mmol/L Tris, pH 7.4) containing 5% skim milk, the samples were incubated with P2Y2 and β-actin primary antibodies overnight at 4 °C; this was followed by exposure to fluorescently labeled secondary antibodies for 1 h at room temperature. Immunoreactive proteins were visualized using an Odyssey infrared imaging system (Li-Cor, Hong Kong, China). Western blotting was repeated at least three times, and one or two representative blots are presented.

### 4.6. YFP Fluorescence Masurement of I^−^ Influx and Flow Cytometry Analysis

Transfected FRT cells were plated in black-walled 96-well plates with transparent plastic bottoms (Corning Costar, Shanghai, China), cultured overnight to confluence, washed three times with PBS, and treated with specified compounds in a final volume of 60 μL. YFP-H148Q/I152L fluorescence was measured using a multi-mode microplate reader FluoStar Optima(BMG LABTECH, Offenburg, Germany) equipped with custom excitation and emission filters (500 nm and 544 nm). The fluorescence intensity in each well was measured for 14 s. In each well, 100 μL of PBS/I^−^ (PBS with 100 mM Cl^−^ replaced by I^−^) was injected by a syringe pump at 2 s after the start of data collection.

The FRT cells that were to be assayed were prepared as a 1 × 10^7^ mL^−1^ cell suspension, and PBS was used as a buffer to exclude background interference. The cell suspensions were assessed using a BD Accuri C6 flow cytometer (BD Biosciences, Beijing, China), and the FL2-A (585 ± 20 nm) channel was selected as the detector for Ano1, whereas the FL1-A (530 ± 15 nm) channel was used to detect Ano1-YFP-H148Q/I152L. A total of 1 × 10^6^ cells were sampled at low speed.

### 4.7. Patch-Clamp

FRT cells were transfected with Ano1 and then inoculated onto slides (Thermo Fisher, Shanghai, China), placed under an inverted fluorescence microscope on day 2, and perfused with electrode external fluid. The resistance of the electrode to water was approximately 4–6 MΩ, and after applying negative pressure to form a 6 GΩ high impedance sealing, the cell membrane at the tip of the electrode was ruptured by exposure to rapid negative pressure to form the whole-cell recording mode. The initial clamp voltage was 0 mV, and after 10 ms of recording, a stepped voltage stimulation was applied from −100 mV to +100 mV, with each increase 20 mV. Each step was recorded for 800 ms, and the voltage became 0 mV after 800 ms. The recording continued for 100 ms (HEKA EPC9, Reutlingen, Germany). The electrode outer solution (bath solution) was 140 mmol/L NMDG-Cl, 2 mmol/L MgCl_2_, 5 mmol/L CaCl_2_, and 10 mmol/L HEPES. The inner electrode solutions (pipette solution) contained zero calcium, 0.6 mmol/L calcium solution, 100 nmol/L NFA, and 0.6 mmol/L calcium solution.

### 4.8. Intracellular Calcium Measurement

When the cells were grown to the appropriate density, the cells were digested using a mixture of 0.25% trypsin and 0.02% EDTA. The digestion was then terminated with the F12 culture medium containing 10% fetal bovine serum, and cell suspensions were prepared. The cells were then collected by centrifugation. The cell suspension was pre-warmed at 37 °C for 5 min, Fura2-AM at a final concentration of 5 μmol/L was added, and the load was incubated at 37 °C for 40 min at a constant temperature while protected from light. The cells were then centrifuged, and the supernatant was discarded. The cells were washed three times with PBS. Changes in intracellular calcium ion concentrations induced by ATP were detected using a multi-mode microplate reader FluoStar Optima (BMG LABTECH, Offenburg, Germany), and the intracellular calcium ion concentration was calculated as the ratio of the fluorescence intensity at 340 nm to 380 nm (F340/F380).

### 4.9. Functional Assessment of Cellular Models for High-Throughput Screening of P2Y2 Modulators

Six columns of a black-walled transparent 96-well plate were filled with 300 μmol/L ATP as the experimental group, whereas the other six columns were filled with sodium iodide PBS buffer as the negative control. The Z-factor values of the 96 wells were then measured. The Z-factor is only related to the repeatability and reliability of the experiment and not to the experimental content. Therefore, it is an important index for high-throughput screening. The Z-factor value can be calculated according to the following formula: Z-factor = 1–3 × (SDsig + SDback)/(Msig-Mback). In this formula, SD represents the standard deviation of the signal or background, and M represents the mean of the signal or background. A general Z-factor value of 0.5 to 1 indicates a high stability for the high-throughput screening model.

### 4.10. Statistical Analysis

All samples’ analyses were repeated three times to ensure the accuracy of the test results. The data were processed using GraphPad Prism8.0 software (San Diego, CA, USA) for the control and experimental groups, and all samples that did not obey normal distribution were analyzed by the rank sum test. The Mann–Whitney test was also used for statistical analysis, and the differences were considered statistically significant at *p* < 0.05.

## Figures and Tables

**Table 1:** The sequences of the primers.

Gene	Primer Sequence (5′-3′)	Product (bp)
β-actin	Forward: GTCGTCGACAACGGCTCC	260
	Reverse: AGGTCTCAAACATGATCTGGGT	
P2Y1	Forward: AGCGTGGCAATCTGGATGTT	472
	Reverse: AGAACATGGCCACAGTCGTG	
P2Y2	Forward: GAGATGACGGGGACCTAAAGAG	365
	Reverse: TCCGTTCCAGTTCTTCACTCG	
P2Y4	Forward: GCACTGGCCTTTGCAAGTTT	439
	Reverse: ACCACAGCAATGGTACGGAG	
P2Y6	Forward: GTCGTTTGGCTGGTTGTGAC	336
	Reverse: GGCTGTCTTGGTGATGTGGA	
P2Y12	Forward: CCCAGCAATCTTTTGGGTGC	422
	Reverse: GTGTTCTCGGCATTGCAGTC	
P2Y13	Forward: CCTCAAGATCGTCGTACCGT	320
	Reverse: TGTGCTTGCTGTCCCTACTT	
P2Y14	Forward: AATCGTGAAGCCCCTTCTGG	373
	Reverse: GAAACAGGCGACAAATGCGA	

## Data Availability

Not applicable.

## Sample Availability

Samples of the compounds are not available from the authors.
